# Reassortant Influenza A(H1N1)pdm09 Virus in Elderly Woman, Denmark, January 2021

**DOI:** 10.3201/eid2712.211361

**Published:** 2021-12

**Authors:** Jakob N. Nissen, Sophie J. George, Charlotte K. Hjulsager, Jesper S. Krog, Xiaohui C. Nielsen, Tina V. Madsen, Klara M. Andersen, Tyra G. Krause, Lasse S. Vestergaard, Lars E. Larsen, Ramona Trebbien

**Affiliations:** Statens Serum Institut, Copenhagen, Denmark (J.N. Nissen, C.K. Hjulsager, J.S. Krog, K.M. Andersen, T.G. Kause, L.S. Vestergaard, R. Trebbien);; University of Copenhagen, Copenhagen (S.J. George, L.E. Larsen);; Zealand University Hospital, Koege, Denmark (X.C. Nielsen, T.V. Madsen)

**Keywords:** influenza, swine flu, influenza A(H1N1)pdm09, Denmark, Orthomyxoviridae, respiratory infections, viral zoonoses, viruses, zoonoses, H1N1, pandemic

## Abstract

A case of human infection with influenza A(H1N1)pdm09 virus containing a nonstructural gene highly similar to Eurasian avian-like H1Nx swine influenza virus was detected in Denmark in January 2021. We describe the clinical case and report testing results of the genetic and antigenic characterizations of the virus.

Human infection with swine influenza A virus (IAV) had not previously been detected in Denmark, but sporadic cases have been reported from other countries (*1*). We report the identification of a case of zoonotic swine influenza infection in Denmark during a low-activity influenza season.

The variant IAV was detected by the National Influenza Center at Statens Serum Institut (Copenhagen, Denmark), as part of routine surveillance. A sputum sample was collected on January 21, 2021, in Zealand, Denmark, from a female patient in her 70s with various concurrent conditions, including a chronic respiratory disease, who was admitted to hospital after 2 days of moderate influenza-like symptoms: fever (39°C), coughing, sore throat, and difficulty breathing. The patient sample was positive for IAV in analyses at the local hospital microbiology laboratory; remaining sample material was submitted to the National Influenza Center, which confirmed it positive for influenza A(H1N1)pdm09 (Appendix).

We performed whole genome sequencing on the virus (*2*), and named it A/Denmark/1/2021 (vH1N1), and submitted to GISAID (https://www.gisaid.org; accession no. EPI_ISL_909652). BLAST (https://blast.ncbi.nlm.nih.gov/Blast.cgi) and phylogenetic analyses revealed that all segments except the nonstructural gene belonged to influenza A(H1N1)pdm09 clade 1A3.3.2 (*3*), which is most similar (97%–98% nt identity) to viruses collected from swine in France and Germany in 2014 and 2015 (Table; Figure). The nonstructural gene was most similar (95%) to Eurasian avian-like H1Nx swine viruses of clade 1C. No segments had a near-exact match to sequences in GenBank or GISAID, and all were distinct from the seasonal vaccine strain, A/Guangdong-Maonan/SWL1536/2019 (Table).

**Table:** Percentage identity similarity between gene and protein segments of influenza virus isolate A/Denmark/1/2021 (vH1N1) from a patient in Denmark and reference viruses from GISAID*

A/Denmark/1/2021 (vH1N1) segment	Identity, %			
	A/swine/Luedinghausen/ 21728/2015†	A/California/07/2009‡	A/Guangdong-Maonan/SWL1536/2019¶	A/swine/Denmark/ 3797–4/2020§
Amino acid				
PB2	98.7	97.5	97.6	100
PB1	99.5	99.3	98.7	99.9
PA	98.9	98.0	98.3	99.6
PA-X	98.7	97.4	97.0	99.6
HA	97.3	92.0	91.9	99.3
NP	99.0	99.0	98.2	100
NA	97.9	95.1	91.9	99.8
M1	98.8	98.4	97.6	100
M2	96.9	96.9	93.8	100
NS1	76.5	77.4	74.7	99.5
NEP	85.1	86.0	85.1	99.2
Nucleotide				
PB2	98.0	96.1	94.7	99.8
PB1	96.8	95.9	93.8	99.2
PA	98.0	96.7	95.4	99.4
HA	97.3	94.4	93.0	99.4
NP	97.4	96.4	94.5	99.4
NA	97.4	96.1	93.7	99.5
MP	97.8	97.4	95.9	99.9
NS	80.2	80.3	80.3	99.8

*GISIAD, https://www.gisaid.org. PB1/PB2, polymerase basic protein 1/2; PA, polymerase acidic protein; HA, hemagglutinin; NP, nucleoprotein; NA, neuraminidase; MP/M1/M2, matrix protein 1/2, NS/NS1; nonstructural protein; NEP, nuclear export protein †GISAID accession no. EPI_ISL_504870. ‡GISAID accession no EPI_ISL_227813. ¶GISAID accession no. EPI_ISL_377080. §GISAID accession no. EPI_ISL_1673668.

**Figure:**
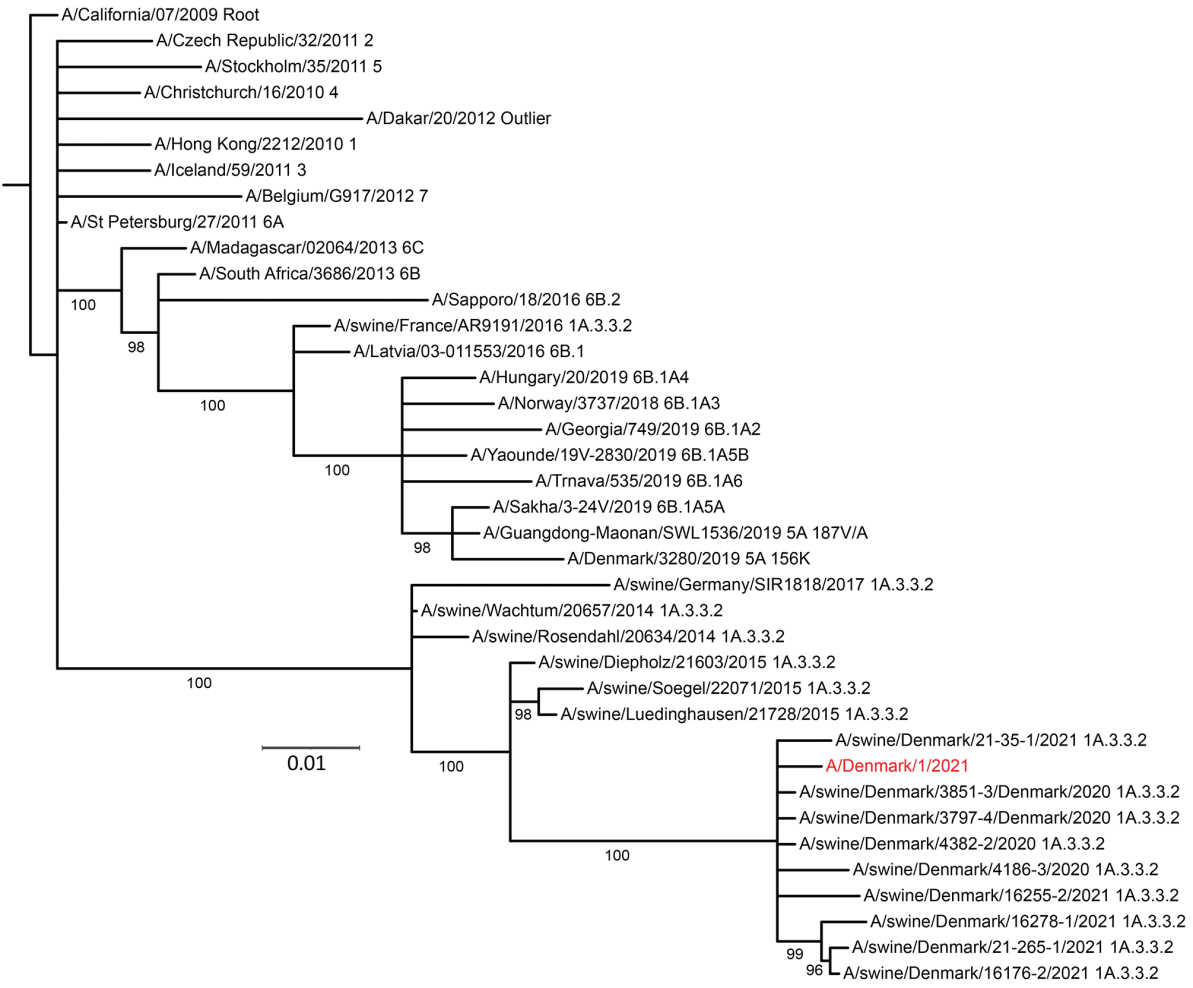
Maximum-likelihood phylogenetic tree of the hemagglutinin gene of influenza virus isolate A/Denmark/1/2021 (vH1N1) from a patient in Denmark (red) and reference viruses. The tree includes closest BLAST matches (https://blast.ncbi.nlm.nih.gov/Blast.cgi), the Denmark swine influenza virus with highest similarity to the case variant virus A/Denmark/1/2021 (indicated in red), and human seasonal reference viruses and is rooted on A/California/07/2009. Leaves are labeled by isolate name and clade designation. Branch labels indicate UFBoot2 bootstrap values. All uncertain branches (bootstrap <95%) have been removed. Scale bar indicates nucleotide substitutions per site.

Because of the suspected swine origin of the case virus, we used whole-genome sequencing to retrospectively analyze 68 IAVs with a hemagglutinin (HA) gene belonging to clade 1A.3.3.2 sampled from swine herds in Denmark during 2020–2021. Nine of the samples, collected April 2020–January 2021 from >7 different herds in different parts of Denmark, including Zealand, contained the same gene constellation as the case virus (98.9%–99.4% nt identity). This finding suggests that the virus from the human case originated from swine in Denmark.

The patient and her husband reside in the countryside, <2 km from a medium-sized farm with finisher pigs. Because of coronavirus disease pandemic restrictions, she had not been in close contact with other persons or been close to the pig farm. Both the patient and her husband, who had no signs of illness, were vaccinated against seasonal influenza in October or November 2020. European General Data Protection Regulation (https://gdpr.eu) restrictions on reporting personally identifiable information prevent revealing additional information about the patient or the farm.

Veterinary authorities in Denmark collected nose swab samples from 68 pigs at the neighboring farm on February 1, 2021, according to standard procedures. All samples tested negative by PCR for IAV. Because of the high prevalence of influenza-positive herds in Denmark, we could not be confident potential seropositive swine were infected by the virus in question, so we did not take blood samples. However, we therefore could not exclude previous virus circulation in the herd, because swabs were taken 11 days after virus detection in the patient. According to the Danish Meteorological Institute, the patient’s residence was downwind of the pig herd most days preceding clinical symptoms.

Most of the case virus genes were derived from influenza A(H1N1)pdm09, which has been circulating in the human population of Denmark since 2009. However, the HA gene is different from that of the strains currently circulating (*4*), and it is therefore difficult to predict the level of immunity in the human population against this virus. Antigenic characterization (*5*) showed no or very poor cross-reactivity to all reference antiserum used for analysis (Appendix Table 1), and the HA gene contained several more mutations at antigenic sites compared with the seasonal vaccine strain (Appendix Figure). Therefore, vaccine effectiveness of the 2020–2021 seasonal influenza vaccine against the variant virus has been assessed as low.

Neuraminidase inhibition tests showed no reduction of oseltamivir or zanamivir inhibition, and the viral genome contains no known antiviral mutations except the V27A mutation in the M2 gene, known from most other H1N1 viruses circulating in human and swine (*6*,*7*). We identified no amino acid changes presumed to be related to increased risk of human infection (*8*), but further in vitro and in vivo analyses are planned to explore this possibility.

Because national coronavirus disease pandemic restrictions limited interpersonal contact, there were only 46 confirmed influenza cases in Denmark during the 2020–2021 season, and transmission of the variant virus was considered negligible. The Danish Patient Authority did not identify any person-to-person swine influenza transmission, and no further public health response measures were enacted.

The effects of the most recent swine influenza pandemic and the extensive diversity and reassortment in swine influenza viruses indicate the obvious zoonotic potential of these viruses (*9*,*10*). Therefore, more attention should be given to routine detection and control of swine influenza viruses.

Appendix Additional information on detection and analysis of swine influenza in Denmark.
