# Supplementary material for: Reassortant Influenza A(H1N1)pdm09 Virus in Elderly Woman, Denmark, January 2021
Source: Emerg Infect Dis. 2021 Dec;27(12):3202–5. doi: 10.3201/eid2712.211361 (PMC8632190; doi:10.3201/eid2712.211361)
Supplement: Appendix — Additional information on detection and analysis of swine influenza in Denmark. [file 21-1361-Techapp-s1.pdf]

Article DOI: https://doi.org/10.3201/eid2712.211361

Reassortant Influenza A(H1N1)pdm09 Virus in Elderly Woman, Denmark, January 2021

Appendix

Methods for Virus Detection and Analysis

We collected sputum samples and analyzed them at the local hospital microbiology laboratory with the SARS-CoV-2 Flu (A+B) & RSV array (CerTest Biotec, https://www.certest.es) on the BD MAX System (Becton Dickinson, https://www.bd.com) and the Xpert Xpress Flu/RSV assay on the GeneXpert XVI system (Cepheid, https://www.cepheid.com). Subsequently, the National Influenza Center analyzed remaining sample materials using in-house real-time reverse transcription PCR to detect the matrix-, H1pdm09-, and N1pdm09-gene segments. For whole genome sequencing, a one-tube RT-PCR approach was used (*1*), and libraries for sequencing on the Miseq platform (Illumina, https://www.illumina.com) were prepared using the Nextera XT DNA preparation kit (Illumina) (*2*).

References

1. Zhou B, Donnelly ME, Scholes DT, St George K, Hatta M, Kawaoka Y, et al. Single-reaction genomic amplification accelerates sequencing and vaccine production for classical and Swine origin human influenza a viruses. J Virol. 2009;83:10309–13. PubMed https://doi.org/10.1128/JVI.01109-09

2. Trebbien R, Pedersen SS, Vorborg K, Franck KT, Fischer TK. Development of oseltamivir and zanamivir resistance in influenza A(H1N1)pdm09 virus, Denmark, 2014. Euro Surveill. 2017;22:30445. PubMed https://doi.org/10.2807/1560-7917.ES.2017.22.3.30445

3. Brownlee GG, Fodor E. The predicted antigenicity of the haemagglutinin of the 1918 Spanish influenza pandemic suggests an avian origin. Philos Trans R Soc Lond B Biol Sci. 2001;356:1871–6. PubMed https://doi.org/10.1098/rstb.2001.1001

Appendix Table 1. Results from antigenic characterization using hemagglutination inhibition assay and microneutralization assay*

Reference virus	A/Guangdong-Maonan/SW1636/2019	A/Victoria/2570/19	A/Wisconsin/588/19	A/Denmark/3280/29	A/Michigan/45/15	A/California/07/09	A/Brisbane/02/2018
Hemagglutinin inhibition test results							
A/Guangdong-Maonan/SW1636/2019	2560						
A/Victoria/2570/19		640					
A/Wisconsin/588/19			1280				
A/Denmark/3280/29				<2560			
A/Michigan/45/15					640		
A/California/07/09						640	
A/Brisbane/02/2018							960
A/Denmark/1/2021	<20	<20	<20	<20	30	<20	<20
Microneutralization results							
A/Michigan/45/15					640	120	
A/California/07/09					40	240	
A/Guangdong-Maonan/SW1636/2019	7680					1280	
A/California/07/09	640					1920	
A/Denmark/1/2021	<20	20	20	20	<20	30	30

*The case variant virus was tested against a panel of reference ferret antisera of A/H1N1pdm09 viruses provided by WHO CC, Francis Crick Institute, UK. The cross-testing of viruses and antisera are indicated with the average titer-value from duplicates. A titer below 20 is considered as no reaction. Thus, in both tests there was no cross-reactivity to the A(H1N1)pdm09 vaccine virus but to some of the other A(H1N1)pdm09 viruses at low levels.

Appendix Table 2. We gratefully acknowledge the authors, originating and submitting laboratories of the sequences from GISAID's EpiFlu Database on which this research is based. All submitters of data may be contacted directly via www.gisaid.org

Segment ID*	Country	Collection date	Isolate-ID	Isolate name	Originating Lab	Submitting Lab	Authors
EPI1785107	Germany	2015 Aug 26	EPI_ISL_504872	A/swine/Soegel/22071/2015		Import from public-domain	Duerrwald, R.; Groth, M.; Krumbholz, A.; Lange, J.; Philipps, A.; Zell, R.
EPI1785091	Germany	2015 May 21	EPI_ISL_504870	A/swine/Luedinghausen/21728/2015		Import from public-domain	Duerrwald, R.; Groth, M.; Krumbholz, A.; Lange, J.; Philipps, A.; Zell, R.
EPI1785075	Germany	2015 Apr 15	EPI_ISL_504868	A/swine/Diepholz/21603/2015		Import from public-domain	Duerrwald, R.; Groth, M.; Krumbholz, A.; Lange, J.; Philipps, A.; Zell, R.
EPI1784875	Germany	2014 Aug 10	EPI_ISL_504843	A/swine/Wachtum/20657/2014		Import from public-domain	Duerrwald, R.; Groth, M.; Krumbholz, A.; Lange, J.; Philipps, A.; Zell, R.
EPI1784859	Germany	2014 Sep 16	EPI_ISL_504841	A/swine/Rosendahl/20634/2014		Import from public-domain	Duerrwald, R.; Groth, M.; Krumbholz, A.; Lange, J.; Philipps, A.; Zell, R.
EPI1201916	Germany	2017 Mar 10	EPI_ISL_304243	A/swine/Germany/SIR1818/2017	Friedrich-Loeffler-Institut	Friedrich-Loeffler-Institut	Harder, Timm C.; Henritzi, Dinah
EPI1080448	France	2016 Nov 14	EPI_ISL_281884	A/swine/France/AR9191/2016		Friedrich-Loeffler-Institut	Henritzi, Dinah; Harder, Timm C.
EPI1365955	Hungary	2018 Dec 17	EPI_ISL_340478	A/Hungary/20/2019	National Public Health Institute	Hungarian National Center of Epidemiology	
EPI1582934	Cameroon	2019 Apr 16	EPI_ISL_388855	A/Yaounde/19V-2830/2019	Centre Pasteur du Cameroun	Crick Worldwide Influenza Centre	
EPI1542971	Georgia	2019 Apr 08	EPI_ISL_377238	A/Georgia/749/2019	National Centre for Disease Control and Public Health	Crick Worldwide Influenza Centre	
EPI748982	Latvia	2016 Feb 29	EPI_ISL_219670	A/Latvia/03–011553/2016	State Agency, Infectology Center of Latvia	Crick Worldwide Influenza Centre	
EPI1543082	Slovakia	2019 Apr 10	EPI_ISL_377294	A/Trnava/535/2019	National Public Health Institute of Slovakia	Crick Worldwide Influenza Centre	
EPI1575087	Russian Federation	2019 Apr 24	EPI_ISL_387098	A/Sakha/3–24V/2019	State Research Center of Virology and Biotechnology	Crick Worldwide Influenza Centre	
EPI1161425	United States	2009 Apr 09	EPI_ISL_227813	A/California/07/2009		Import from public-domain	Tan, G.; Pickett, B.; Fedorova, N.; Amedeo, P.; Isom, R.; Hu, L.; Christensen, J.; Miller, J.; Durbin, A.; Arumemi, F.; Williams, T.; Bao, Y.; Sanders, R.; Zhdanov, S.; Kiryutin, B.; Lipman, D.J.; Tatusova, T.; Hatcher, E.; Wang, J.
EPI1542570	China	2019 Jun 17	EPI_ISL_377080	A/Guangdong-Maonan/SWL1536/2019	WHO Chinese National Influenza Center	WHO Chinese National Influenza Center	Xiaoxu, Zeng; Xiyan, Li; Weijuan, Huang; Lei, Yang; Dayan, Wang
EPI466580	Madagascar	2013 May 24	EPI_ISL_145424	A/Madagascar/02064/2013	Institut Pasteur de Madagascar	National Institute for Medical Research	

Segment ID*	Country	Collection date	Isolate-ID	Isolate name	Originating Lab	Submitting Lab	Authors
EPI417118	Belgium	2012 Dec 07	EPI_ISL_134397	A/Belgium/G917/2012	Scientific Institute of Public Health	National Institute for Medical Research	
EPI346697	Iceland	2011 Mar 24	EPI_ISL_99924	A/Iceland/59/2011	Landspitali - University Hospital	National Institute for Medical Research	
EPI417122	Senegal	2012 Dec 09	EPI_ISL_134399	A/Dakar/20/2012	Institut Pasteur de Dakar	National Institute for Medical Research	
EPI770076	Japan	2016 Feb 01	EPI_ISL_223792	A/SAPPORO/18/2016	Sapporo City Institute of Public Health	National Institute of Infectious Diseases	Takashita, Emi; Fujisaki, Seiichiro; Shirakura, Masayuki; Watanabe, Shinji; Odagiri, Takato
EPI347564	Sweden	2011 Nov 22	EPI_ISL_100460	A/Stockholm/35/2011		Public Health Agency of Sweden	
EPI705858		2016 Feb 09	EPI_ISL_210175	A/Christchurch/16/2010 NIB-74xp (13/202)	National Institute for Biologic Standards and Control (NIBSC)	National Institute for Biologic Standards and Control	Nicolson, Carolyn
EPI279895	Hong Kong (SAR)	2010 Jul 16	EPI_ISL_79623	A/Hong Kong/2212/2010	Government Virus Unit	National Institute for Medical Research	
EPI319447	Czech Republic	2011 Jan 18	EPI_ISL_90718	A/Czech Republic/32/2011	National Institute of Public Health	National Institute for Medical Research	
EPI319527	Russian Federation	2011 Feb 14	EPI_ISL_90760	A/St. Petersburg/27/2011	WHO National Influenza Centre Russian Federation	National Institute for Medical Research	
EPI466630	South Africa	2013 Jun 10	EPI_ISL_145449	A/South Africa/3686/2013	National Institute for Communicable Disease	National Institute for Medical Research	
EPI1393451	Norway	2018 Nov 27	EPI_ISL_347404	A/Norway/3737/2018	WHO National Influenza Centre	Crick Worldwide Influenza Centre	

*All segments are hemagglutinin.

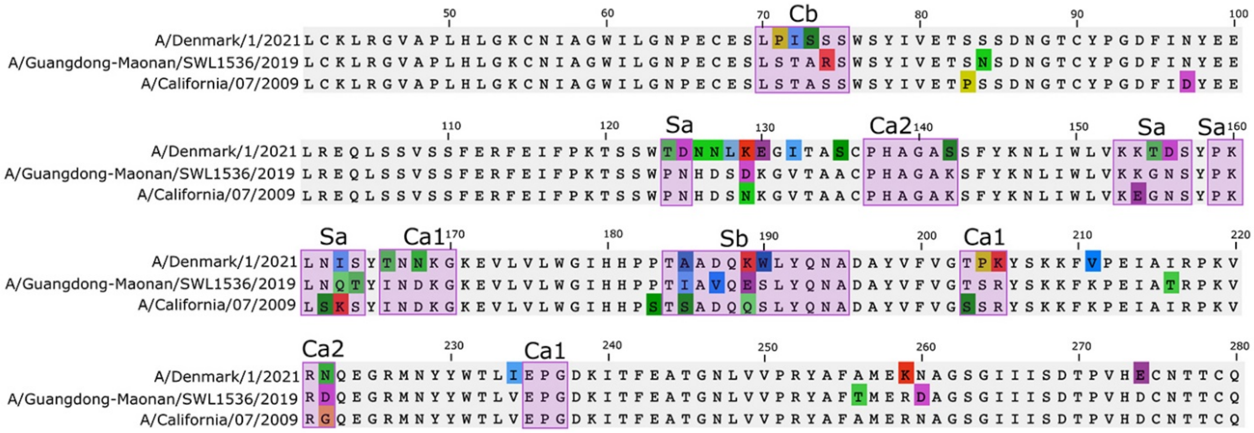

Appendix Figure. Alignment of HA amino acid sequences of case variant virus A/Denmark/1/2021, A/California/07/2009, and seasonal vaccine strain A/Guangdong-Maonan/SWL1536/2019. The alignment is shown with H1 numbering starting after the signal peptide, and the antigenic sites Ca1, Ca2, Cb, Sa, Sb, as defined by Brownlee and Fodor (*3*), are indicated with shaded boxes. Mutations relative to each other have been highlighted.
